# Cellular Pharmacological Effects of the Traditional Japanese Kampo Medicine Yokukansan on Brain Cells

**DOI:** 10.3389/fphar.2017.00655

**Published:** 2017-09-20

**Authors:** Kazushige Mizoguchi, Yasushi Ikarashi

**Affiliations:** Tsumura Kampo Research Laboratories, Kampo Research & Development Division, Tsumura & Co. Ibaraki, Japan

**Keywords:** yokukansan, neuron, astrocyte, microglia, blood–brain barrier, neuroprotection

## Abstract

Yokukansan (YKS) is a traditional Japanese Kampo medicine currently used for the treatment of the behavioral psychological symptoms associated with dementia (BPSD), which is frequently problematic in neurodegenerative disorders such as Alzheimer’s disease. Regarding the pharmacological mechanisms underlying its efficacy, we recently reviewed the multiple effects of YKS on the neurotransmitter systems (e.g., glutamatergic, serotonergic, dopaminergic, cholinergic, GABAergic, and adrenergic neurotransmission) in various brain regions that are related to the psychological, emotional, cognitive, or memory functions. These multiple effects are thought to be caused by multiple components included in YKS. In addition, YKS exhibits various effects on brain cells (i.e., neurons, glial cells including astrocytes, oligodendrocytes, and microglial cells, and endothelial cells). In this review, we summarize recent evidence demonstrating the cellular pharmacological effects of YKS on these brain cells, and discuss the current understanding of its efficacy and mechanism. In particular, YKS maintains the neuronal survival and function by multiple beneficial effects, including anti-apoptosis, anti-oxidation, anti-endoplasmic reticulum stress, and neurogenesis. YKS also acts on glial cells by: facilitating the transport of glutamate into astrocytes; promoting the proliferation and differentiation of oligodendrocytes; and enhancing the anti-inflammatory properties of microglial cells. These glial effects are thought to support neuronal functioning within the brain. Various ingredients involved in these effects have been identified, some of which can pass through the artificial blood–brain barrier without disrupting the endothelial tight junctions. This multitude of interactive effects displayed by YKS on neuronal and glial cells is suggested to be involved in the multitude of neuropsychopharmacological actions of YKS, which are related to the improvement of BPSD.

## Introduction

Recently, the beneficial clinical efficacy of traditional Japanese Kampo medicine has been reported when applied for the treatment of diseases that are insufficiently cured with Western medical approaches. Yokukansan (YKS) originates from the traditional Chinese medicine *Yi-Gan-San* and is one of the Kampo prescriptions approved by the Japanese Ministry of Health, Labor, and Welfare as a remedy for neurosis, insomnia, as well as night crying and irritability for children. It is composed of seven dried medicinal herbs: *Atractylodes lancea rhizome* (*Atractylodes lancea* De Candolle, Compositae); *Poria sclerotium* (*Poria cocos* Wolf, Polyporaceae); *Cnidium rhizome* (*Cnidium officinale* Makino, Umbelliferae); *Uncaria hook* (*Uncaria rhynchophilla* Miquel, Rubiaceae); *Japanese Angelica root* (*Angelica acutiloba* Kitagawa, Umbelliferae); *Bupleurum root* (*Bupleurum falcatum* Linné, Umbelliferae); and *Glycyrrhiza* (*Glycyrrhiza uralensis* Fisher, Leguminosae). To date, various ingredients responsible for the neuropsychopharmacological actions of YKS have been identified (Ikarashi and Mizoguchi, 2016; Mizoguchi and Ikarashi, 2017).

Yokukansan is currently used clinically to treat behavioral and psychological symptoms associated with dementia (BPSD), such as hallucinations, agitation, and aggressiveness in patients with Alzheimer’s disease, dementia with Lewy bodies, vascular dementia, and other forms of senile dementia; it has been demonstrated to be an effective and well-tolerated treatment for patients with BPSD without severe adverse effects (Iwasaki et al., 2005, 2012; Mizukami et al., 2009; Monji et al., 2009; Okahara et al., 2010; Nagata et al., 2012; Matsuda et al., 2013). *In vivo* and *in vitro* basic research conducted to clarify the efficacy and mechanisms of YKS have demonstrated that it ameliorates various symptoms, including cognitive deficits, BPSD-like behaviors, and non-BPSD-like behaviors (e.g., tardive dyskinesia, neuropathic pain, allergy, morphine tolerance, and physical dependency) (Mizoguchi and Ikarashi, 2017). Additionally, regulatory effects on multiple neurotransmitter systems, including glutamatergic, serotonergic, cholinergic, dopaminergic, adrenergic, and gamma-aminobutyric acid (GABA)ergic neurons are suggested to be the neuropharmacological mechanisms underlying the efficacy of YKS (Ikarashi and Mizoguchi, 2016). Moreover, brain regions (e.g., cerebral cortex, amygdala, hippocampus, striatum, thalamus, hypothalamus, corpus callosum, medulla-pons, and spinal cord) which are related to behavioral, psychological, cognitive, or learning and memory functions, are the suggested target regions that contribute to the psychopharmacological efficacy of YKS (Mizoguchi and Ikarashi, 2017).

The brain is primarily composed of two broad classes of cells: neurons and glial cells (Hansson and Rönnbäck, 2003; Kettenmann and Verkhratsky, 2011). Neurons comprise only 10% of the brain cells but are the most important cells involved in information processing (i.e., the neuronal network for processing information is formed by complex interactions and communication between the neurons) (Mariani, 1983; Hashimoto and Kano, 2003; Nakayama et al., 2005). On the other hand, 90% of brain cells are comprised of several types of glial cells (e.g., astrocytes, oligodendrocytes, and microglia), which perform a number of critical functions, including structural and metabolic support, as well as insulation. In addition, two-way communication between neurons and glial cells is essential for the maintenance of the axonal condition, synaptic transmission, and information processing, and thus, is required for normal functioning of the nervous system (Vernadakis, 1996; Gomes et al., 2001; Fields and Stevens-Graham, 2002; Kettenmann and Verkhratsky, 2011).

The fundamental cause of neurodegenerative diseases is neuronal death, which is induced by pathogenic factors, such as glutamate excitotoxicity, amyloid β (Aβ) neurotoxicity, neuroinflammation, oxidative stress, and apoptotic signaling. Therefore, one strategy to treat these diseases is via protection of neuronal death, focusing on neurons (e.g., blockade of glutamate receptors and the removal of Aβ). Additionally, recent studies have indicated the importance of the neuron-glia interaction. For example, excessive glutamate in the synaptic cleft causes excitotoxicity in post-synaptic neurons, and is taken up by astrocytes through the glutamate transporters expressed on their plasma membrane (Kawakami et al., 2009). This can be useful as an alternative strategy to treat various neurodegenerative diseases. Thus, pharmacological approaches that target neurons, as well as glial cells are ideal to prevent the neuronal death related to such diseases.

To date, basic research investigating the neuropsychopharmacological actions of YKS have reported the involvement of brain cells and its underlying mechanisms. These have suggested that YKS has protective and reparative effects against neuronal abnormalities (Tanaka and Mizoguchi, 2009; Hiratsuka et al., 2010; Kawakami et al., 2011a,b; Kanno et al., 2013, 2014, 2015; Kubota et al., 2013), astrocytes (Kawakami et al., 2009, 2010), oligodendrocytes (Morita et al., 2014; Ueki et al., 2014; Shimizu et al., 2015), and microglial cells (Furuya et al., 2013; Liu et al., 2014). In addition, some of the active ingredients in YKS were examined regarding their permeability using an artificial blood–brain barrier (BBB) model that was constructed using endothelial cells (Imamura et al., 2011; Tabuchi et al., 2012). In this review, we summarized basic research from the last decade that demonstrates the effects of YKS on these brain cells, and discuss the current understanding of its efficacy and mechanisms.

## Methodology

The authors searched the term “yokukansan” in PubMed, Scopus, and ScienceDirect, and a total of 171 articles were selected and used for this review. Inclusion criteria: articles describing the cellular effects of YKS between 2005 and 2017 included in PubMed, Scopus, or ScienceDirect. Exclusion criteria: articles published in a language other than English.

## Effects of YKS on Brain Cells

### Neuron

Neurons are usually considered the most important cells in the brain. Each neuron consists of a cell body, an axon, and neurites, constructing a neural network of information via communication between neurons through the axon and neurites (Mariani, 1983; Hashimoto and Kano, 2003; Nakayama et al., 2005). Several neural networks are closely involved in behavioral performance, psychological status, and emotion, as well as thinking, learning, and memory. Therefore, neuronal death induced by pathogenic factors disrupts numerous brain functions. Neuronal death commonly occurs in the brains of individuals with several neurodegenerative diseases (e.g., Alzheimer’s, Parkinson’s, and cerebrovascular disease), and subsequently causes multiple cognitive deficits, memory impairment, and BPSD (Iwasaki et al., 2012; Nagata et al., 2012; Matsuda et al., 2013).

**FIGURE 1:**
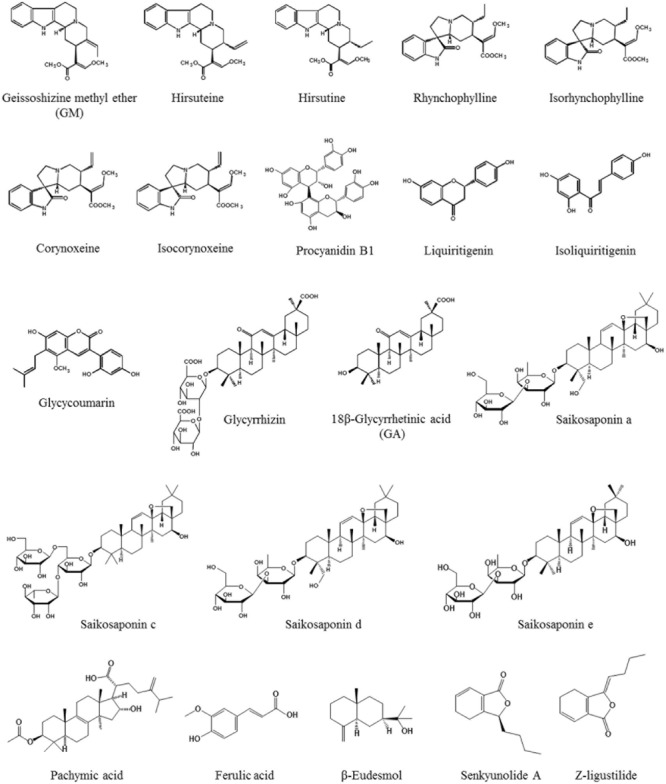
Chemical structures of active ingredients included in YKS.

Yokukansan has been reported to ameliorate cognitive, learning, and memory disturbances, in addition to the accompanied BPSD-like behaviors in various rodent models of dementia (Ikarashi et al., 2009; Makinodan et al., 2009; Sekiguchi et al., 2009, 2011; Tabuchi et al., 2009; Fujiwara et al., 2011; Mizoguchi et al., 2011; Furuya et al., 2013; Nogami et al., 2013; Uchida et al., 2013; Liu et al., 2014). As one of the mechanisms underlying these ameliorations, the neuroprotective activity of YKS has been suggested to be involved. In addition, several active ingredients have been identified, including geissoschizine methyl ether (GM), hirsuteine, hirsutine, and procyanidin B1 in *Uncaria hook*, and glycycoumarin in *Glycyrrhiza*, all of which protect glutamate-induced neuronal or PC12 cell death (Kawakami et al., 2011a,b; Kanno et al., 2014) (**Figure 1**). Excessive glutamate induces neurotoxicity by both over-stimulating the *N*-methyl-D-aspartate receptor and inhibiting the cysteine/glutamate antiporter system Xc^-^ that facilitates the synthesis of antioxidant glutathione (GSH) for cytoprotection (Choi, 1988; Murphy et al., 1990; Schubert et al., 1992; Froissard and Duval, 1994; Pereira and Oliveira, 2000; Penugonda et al., 2005; Edwards et al., 2007; Lo et al., 2008). The cytoprotective effects of YKS may involve both mechanisms, but the latter may be predominantly involved (Kawakami et al., 2011a; Kanno et al., 2014).

The system Xc^-^ consists of two protein components: (1) the xCT protein responsible for transport activity; and (2) the 4F2 heavy chain (4F2hc) necessary for membrane localization of the heterodimer (Lo et al., 2008; Conrad and Sato, 2012; Lewerenz et al., 2012, 2013). Interestingly, during the progression of cell death by over-glutamate exposure for 24 h, the gene expression of xCT and 4F2hc increased at early stage (6 h following glutamate exposure), for which no cell death was observed (Kanno et al., 2014). The cell survival at this stage was thought to be due to the enhanced defense system by overexpressing system Xc^-^ genes. However, the prolonged exposure of glutamate for 24 h resulted in cell death by the breakdown of this defense system. YKS and four *Uncaria hook* ingredients (e.g., GM, hirsuteine, hirsutine, and procyanidin B1) protected glutamate-induced cell death at 24 h combined with the further enhancement of both the gene expression of xCT and 4F2hc at 6 h, and subsequent GSH production. This effect was counteracted by co-treating the system Xc^-^ inhibitor (S)-4-carboxyphenylglycine. Thus, the enhancement of system Xc^-^ gene expression by these factors has been suggested to contribute to the cytoprotective efficacy of YKS by preserving the cellular antioxidant ability (Kanno et al., 2014). On the other hand, the *Glycyrrhiza* ingredient, glycycoumarin, demonstrated a potent cytoprotective effect by increasing the GSH level without the induction of the xCT or 4F2hc gene expression (Kanno et al., 2014). Therefore, this aspect is thought to be the direct activator against GSH, not via system Xc^-^. The transcription factor Nrf2 for GSH synthesis may be potentially related to the direct effect of YKS (Bridges et al., 2012). YKS and three *Uncaria hook* ingredients (e.g., GM, hirsuteine, and hirsutine) also inhibited cystine deficiency- or system Xc^-^ inhibitor-induced cell death by increasing the level of GSH (Kawakami et al., 2011b). Therefore, these ingredients protected cell death by directly increasing the intracellular GSH levels even in the absence of the antiporter. The string of these experimental results suggests that GSH-mediated cytoprotection by YKS is thought to be mediated by individual ingredients by three mechanisms: (1) procyanidin B1 having GSH synthetic effect via system Xc^-^; (2) glycycoumarin having a direct GSH synthetic effect not via system Xc^-^; and (3) GM, hirsuteine, and hirsutine both exhibiting GSH synthetic effects.

In addition, YKS has been demonstrated to prevent endoplasmic reticulum (ER) stress-induced apoptosis in SK-N-SH human neuroblastoma cells and Neuro-2a mouse neuroblastoma cells by both upregulating chaperone protein GRP78/Bip expression for unfolded protein repair and inhibiting CCAAT/enhancer-binding protein (C/EBP) homologous protein (CHOP) expression for the induction of apoptosis (Hiratsuka et al., 2010). Ferulic acid in Cnidium rhizome is also suggested to be an active ingredient in YKS. Moreover, YKS inhibits Aβ oligomer-induced apoptosis in primary cultured cortical neurons by suppressing apoptosis effector caspase-3/7 activity (Tateno et al., 2008; Kanno et al., 2013); glycycoumarin and procyanidin B1 are presumed to contribute to this neuroprotection (Kanno et al., 2015).

Yokukansan has also been reported to exert neuroplasticity-facilitating effects. YKS increased bromodeoxyuridine-labeled cells in the hippocampal dentate gyrus and prefrontal cortex in young and aged rats, respectively, suggesting that YKS enhances the neurogenesis and migration of neural stem cells (Tanaka and Mizoguchi, 2009). Similar effects have been reported in schizophrenia model Gunn rats (Furuya et al., 2013). YKS also decreased accumulation of aggrecan, a major chondroitin sulfate proteoglycan, which suppresses neurite outgrowth and cell migration, in the prefrontal cortex and hippocampus of aged rats (Tanaka and Mizoguchi, 2009). In addition, YKS promoted neurite outgrowth of PC12 cells (Kubota et al., 2013) or neuronal protection (Doo et al., 2010) through the activation of nerve growth factor-like phosphorylation and activation of protein kinase and lipid kinase pathways, such as extracellular signal-regulated kinase 1/2 (ERK1/2) and phosphatidylinositol 3-kinase/Akt (PI3K/Akt) (Tsuji et al., 2001; Vaudry et al., 2002; Chen et al., 2012). β-Eudesmol derived from *Atractylodes lancea* rhizome is suggested to be one of candidates involved in the observed neurotrophic effects (Obara et al., 2002; Kubota et al., 2013). Taken together, these findings suggest that YKS may promote neuroplasticity via neurogenesis, including proliferation and differentiation, as well as neurite outgrowth even in the aged brain.

Recently, saikosaponin d, a major triterpenoid saponin derived from Bupleurum root, has been reported to facilitate autophagy, an important cellular process that controls cells in a normal homeostatic state by recycling nutrients to maintain cellular energy levels for cell survival via the turnover of proteins and damaged organelles (Wong et al., 2013a,b; Law et al., 2014). In the future, it should be clarified whether YKS containing this ingredient participates in a phenomenon related to autophagy.

Finally, as one of the important neuropharmacological effects of YKS, the regulatory mechanisms for various neurotransmitter systems (i.e., serotonergic, glutamatergic, cholinergic, dopaminergic, adrenergic, and GABAergic systems) have been reported. These aspects have recently been reviewed (see for more information: Ikarashi and Mizoguchi, 2016).

### Astrocyte

Cognitive deficit and BPSD are thought to be associated with the dysfunction of neural systems and neuronal degeneration in the brain. In several animal models of dementia, increased extracellular levels of glutamate have been demonstrated in the brain (Harkany et al., 2000; Todd and Butterworth, 2001; Behrens et al., 2002; Han et al., 2008). It is well-known that increased glutamate levels induce the excitation of post-synaptic neurons; however, excessive glutamate exacerbates excitotoxic neuronal death (Choi, 1988; Cheung et al., 1998). Under normal physiological conditions, glutamate neurotransmission to post-synaptic receptors is terminated by the clearance of glutamate from the synaptic cleft through its transporter proteins located on the plasma membrane of neurons and astroglial cells to prevent an overload of glutamate to neurons. In particular, astrocytes play an important role in the efficient removal of glutamate from the extracellular space via two glutamate transporters: (1) the glutamate aspartate transporter (GLAST); and (2) glutamate transporter 1 (GLT-1) (Kanai et al., 1993; Schlag et al., 1998). Therefore, a failure of this glutamatergic neurotransmission clearance system induces the hyper-excitation of neurons and neuronal death, which cause BPSD and cognitive deficits.

A thiamine deficiency induced memory disturbances and BPSD-like symptoms, including anxiety and aggressive behavior in rats (Ikarashi et al., 2009; Iizuka et al., 2010). The severe degeneration of astrocytes and elevated extracellular glutamate were observed in their brain. These findings suggest that a thiamine deficiency increases extracellular glutamate levels by reducing the level of glutamate uptake into astrocytes through the glutamate transporters. Moreover, YKS prevented memory disturbances, BPSD-like behaviors, astrocyte degeneration, and increased glutamate levels resulting from thiamine deficiency (Ikarashi et al., 2009). This suggests that YKS prevents these dysfunctions via an improvement of reduced glutamate uptake. Indeed, YKS ameliorated the thiamine deficiency-induced decrease in both the glutamate uptake into astrocytes *in vitro* and the concomitant reduction in the mRNA or protein expressions of GLAST and GLT-1 (Kawakami et al., 2009). Protein kinase C inhibition has been known to facilitate the glutamate transport activity (Conradt and Stoffel, 1997; Gonzalez et al., 1999; Hazell et al., 2003). Since glycyrrhizin and 18β-glycyrrhetinic acid (GA) were shown to suppress protein kinase C activity in cultured astrocytes (Kawakami et al., 2009, 2010), these ingredients are likely responsible for the ameliorative effects of YKS on the dysfunction of glutamate transport in astrocytes. More recently, YKS was demonstrated to alleviate the decrease in GLT-1 protein expression in the hippocampus of stress-maladaptive mice (Miyagishi et al., 2017). Microautoradiography using normal rat hippocampal slices revealed that [^3^H]GA signals were distributed in small non-neuronal cells resembling astrocytes (Mizoguchi et al., 2014). Furthermore, an immunohistochemical analysis revealed that the immunoreactivity for 11β-hydroxysteroid dehydrogenase type-1, a defined molecule recognized by GA (Irie et al., 1992), was detected in the astrocytes of the hippocampus (Mizoguchi et al., 2014). These results suggest that the pharmacological actions of GA may be related to 11β-hydroxysteroid dehydrogenase type-1 in astrocytes. Thus, YKS is thought to control the external environment of neurons by removing excess glutamate via improvement of astrocyte dysfunction.

### Oligodendrocyte

Oligodendrocytes are cells forming the myelin sheath surrounding the axons in a concentric manner within the central nervous system. It facilitates the rapid saltatory conduction of electrical impulses along the axon (Keirstead and Blakemore, 1999; Morrell and Quarles, 1999). In normal brains, although demyelination occurs, it is spontaneously repaired via remyelination mechanisms mediated by oligodendrocyte precursor cells (OPCs) (Baer et al., 2009). However, in patients with multiple sclerosis, this recovery process ultimately fails, and persistent demyelination with the subsequent axonal loss results in the progression of irreversible functional deficits (Keirstead and Blakemore, 1999; Mi et al., 2009). A similar level of demyelination has been demonstrated in the brains of aged rats (Kobayashi et al., 2003), conditions of hypoxia (Akundi and Rivkees, 2009), thiamine-deficient Wernicke’s encephalopathy (Hazell et al., 1998), and Alzheimer’s disease (Zhan et al., 2014).

We have previously demonstrated that YKS ameliorated not only memory dysfunction and BPSD-like behaviors, but also the demyelination and degeneration of oligodendrocytes in thiamine-deficient rat brain (Ikarashi et al., 2009; Iizuka et al., 2010). These findings led us to speculate that the effectiveness of YKS may be related to the repair of demyelination by regulating the function of oligodendrocytes.

Consequently, to clarify whether YKS affects oligodendrocytes, the effects on the proliferation and differentiation of oligodendrocytes were directly examined using high-purity mouse cortical OPCs (Ueki et al., 2014). YKS increased the number of OPCs in the proliferation stage and differentiated cells in the differentiation stage of the culture period. *Uncaria hook-*derived GM showed similar effect to that observed by YKS. This finding suggested that YKS promotes the proliferation and differentiation of oligodendrocytes, for which GM is one of active ingredients responsible for this effect.

Subsequently, Morita et al. (2014) examined the effect of GM on the oligodendrocyte lineage in the medial prefrontal cortex of cuprizone-fed mice, an animal model of schizophrenia. As a result, GM treatment increased mature oligodendrocytes, but there was no effect against immature oligodendrocytes. These results suggest that GM exhibits a specific differentiation-promoting effect on OPCs and likely functions as a remyelination promoter in cuprizone mice.

Taken together, these findings suggest that *Uncaria hook*-derived alkaloid GM contained in YKS may enhance both the proliferation of OPCs and their differentiation into oligodendrocytes. Unfortunately, the mechanism of OPC lineage progression by GM has not been clarified. As a possible mechanism, we consider the involvement of extracellular signal regulated kinase pathway or protein kinase C, which is known to be related to OPC differentiation (Baron et al., 1998; Baer et al., 2009). This is due to the activation of ERK 1/2 and PI3K/Akt (Kubota et al., 2013) by YKS as described in “Neuron,” and the inhibition of protein kinase C activity (Kawakami et al., 2010) as described in “Astrocyte.” A detailed examination to clarify the molecular mechanism is necessary in the future.

### Microglia

Microglial cells are found in all regions of the brain and spinal cord. They are mobile within the brain and proliferate when the brain is subjected to damage or infection (Gomes et al., 2001). These microglial responses generally serve to minimize damage and protect the neurons. Nevertheless, the strong activation of microglia promotes tissue injury rather than repair (Boje and Arora, 1992; Brodal, 2010). Several studies have suggested that the inflammation associated with activated microglia is detrimental to the survival of hippocampal neurons (Monje et al., 2003; Kohman and Rhodes, 2013).

Recently, YKS has been reported to attenuate cerebral ischemia-induced microglial inflammatory response and subsequent neuronal apoptosis in the hippocampal CA1 region of gerbils (Liu et al., 2014). Moreover, YKS inhibited microglial activation, and promoted neurogenesis in the hippocampal dentate gyrus of Gunn rats (Furuya et al., 2013). These results suggest that the ameliorative effect of YKS on cognitive deficits is mediated by promoting neurogenesis associated with the suppression of activated microglia. At the present time, active ingredients responsible for this YKS action remain unclear. However, YKS is included in several ingredients exerting an anti-inflammatory effect as follows: *Atractylodes lancea rhizome*-derived β-eudesmol (Seo et al., 2011), *Poria sclerotium*-derived pachymic acid (Nukaya et al., 1996), *Cnidium rhizome*-derived senkyunolide A and Z-ligustilide (Or et al., 2011), *Cnidium rhizome* and *Japanese angelica root*-derived ferulic acid (Cheng et al., 2008), *Uncaria hook*-derived rhynchophylline (Yuan et al., 2009; Song et al., 2012) and isorhynchophylline (Yuan et al., 2009), *Bupleurum root*-derived saikosaponin a, c, d, and e (Lu et al., 2012), and *Glycyrrhiza*-derived liquiritigenin (Kim et al., 2008) and GA (Khaksa et al., 1996) (**Figure 1**). In the future, the relationship between these ingredients in relation to anti-microglial activation should be clarified.

Microglial cells also release glutamate through gap junction hemichannels when they are activated, which contribute to excitotoxic neuronal death. This is known to result in non-cell-autonomous neuronal death in neurodegenerative diseases (Lobsiger and Cleveland, 2007). A gap junction hemichannel blocker based on GA that has astroglial glutamate uptake and anti-inflammatory effects has been demonstrated to suppress activated microglia and macrophage-mediated neuronal death in rodent models of transient ischemia brain injury (Takeuchi et al., 2008) and experimental autoimmune encephalomyelitis (Jin et al., 2009). A similar suppressive effect of the blocker has been reported in mouse models of amyotriphic lateral sclerosis and Alzheimer’s disease (Takeuchi et al., 2011).

### Endothelial Cell

Brain capillary endothelial cells interact with other neighboring cells, astrocytes, pericytes, perivascular microglia, and neurons (Joó, 1996; Deli et al., 2005; Abbott et al., 2006; Cecchelli et al., 2007). In particular, the cross-talk between the cells of a neurovascular unit is crucial for the formation and maintenance of a functional BBB (Abbott et al., 2006; Zlokovic, 2008). Recently, an artificial BBB model constructed from primary cultured rat cells, including brain microvessel endothelial cells, pericytes, and astrocytes, which mimic the anatomical condition *in vivo*, has been used for the evaluation of BBB permeability of psychopharmaceuticals (Nakagawa et al., 2007, 2009).

Using this model, the BBB permeability of several ingredients in YKS was examined. Among the seven alkaloids in *Uncaria hook* (i.e., GM, hirsutine, hirsuteine, rhynchophylline, isorhynchophylline, corynoxeine, and isocorynoxeine), GM was associated with the highest permeability rate at 27.3%, while the reference drug carbamazepine was 52.0% (Imamura et al., 2011). Additionally, among the triterpenes (e.g., glycyrrhizin and GA) and flavonoids (e.g., liquiritin, liquiritigenin, isoliquiritn, and isoliquiritigenin) in *Glycyrrhiza*, liquiritigenin demonstrated the highest permeability rate at 33.4% (Tabuchi et al., 2012); GA that acts on astrocytes to facilitate glutamate uptake was 13.3%. Indeed, GM and GA were detectable in the brains after an oral administration of YKS in rats (Imamura et al., 2011; Tabuchi et al., 2012). It is important to note that not all of these ingredients affected the endothelial tight junctions, whose disruption causes greater passage of molecules through the BBB, such as lipopolysaccharides (De Vries et al., 1996; Gaillard et al., 2003; Veszelka et al., 2007).

## Conclusion

**FIGURE 2:**
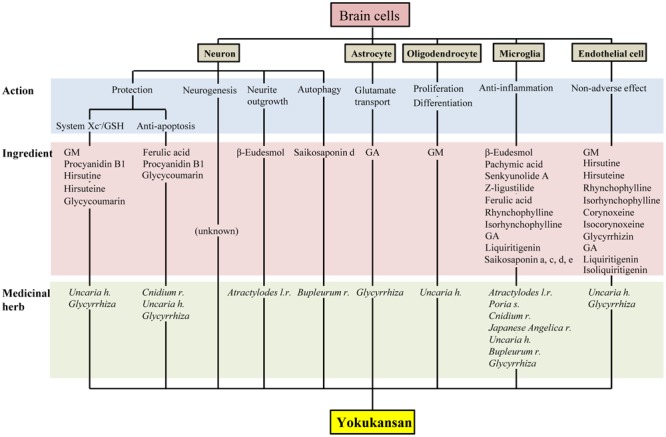
Multiple pharmacological actions of yokukansan on brain cells, as well as the responsible constituent medicinal herbs and ingredients. GSH, glutathione; GA, 18β-glycyrrhetinic acid; GM, geissoschizine methyl ether; *h, hook; l, lancea; r, rhizome; s, sclerotium*.

Yokukansan has been shown to ameliorate abnormal behaviors, psychological symptoms, cognitive deficits, as well as learning and memory impairment in several animal models of dementia and BPSD via multiple neuropsychopharmacological mechanisms (Ikarashi and Mizoguchi, 2016; Mizoguchi and Ikarashi, 2017). The brain regions responsible for these mechanisms are composed of brain cells consisting primarily of neurons and glial cells (i.e., astrocytes, oligodendrocytes, and microglial cells). In this review, we introduced the current findings related to the pharmacological effects of YKS against brain cells, which are summarized in **Figure 2**. Accumulated evidence suggests that YKS has neuroprotective properties that are mediated by increasing the anti-oxidant ability of neurons via enhancing system Xc^-^ function, anti-ER stress effects, and anti-apoptotic effects. YKS is also suggested to exhibit neuroplasticity-related effects, including neurogenesis and neurite outgrowth. Regarding non-neuronal cells, YKS normalizes reduced glutamate transporter function in astrocytes, which mediates removal of excess glutamate in the synaptic cleft, and protects glutamate-induced excitotoxicity for neurons. YKS is thought to play an important role in the maintenance of neuronal signal conduction by both the proliferation of OPCs and their differentiation into oligodendrocytes within the brains of individuals affected by neurodegenerative diseases. YKS also suppresses excessive activation of microglia in brains affected by inflammation, ischemia/reperfusion injury, and schizophrenic events.

As described above, accumulating evidence suggests that YKS might maintain neuronal function by comprehensively acting on neurons as well as various glial cells that surround them. **Figures 1, 2** present the candidates estimated to be involved in herbal medicines and the active ingredients that act on various cell types in the brain. This information facilitates our understanding of a variety of neuroprotective effects mediated by the multi-component drug, YKS, which is a useful strategy for preventing neuronal death, a fundamental cause of multiple neurodegenerative diseases.

## Author Contributions

KM and YI cooperatively corrected the findings, and prepared the manuscript.

## Conflict of Interest Statement

The authors are employees of Tsumura & Co. The authors declare that, except for income received from the employer, no financial support or compensation has been received from any individual or corporate entity and no conflict of interest exists.
